# Strong, tough and bio-degradable polymer-based 3D-ink for fused filament fabrication (FFF) using WS_2_ nanotubes

**DOI:** 10.1038/s41598-020-65861-w

**Published:** 2020-06-01

**Authors:** Hila Shalom, Sergey Kapishnikov, Vlad Brumfeld, Naum Naveh, Reshef Tenne, Noa Lachman

**Affiliations:** 1 Department of Materials Science and Engineering, Faculty of Engineering, Tel-Aviv University, Ramat Aviv, Tel Aviv, 6997801 Israel; 2 Department of Materials and Interfaces, Weizmann Institute of Science, Rehovot, 76100 Israel; 3 Department of Chemical Research Support, Weizmann Institute of Science, Rehovot, 76100 Israel; 4 Polymers and Plastics Engineering Department, Shenkar College of Engineering, Design and Art, Ramat-Gan, Israel

**Keywords:** Mechanical properties, Design, synthesis and processing, Composites

## Abstract

WS_2_ inorganic nanotubes (WS_2_-NT) have been incorporated into Polylactic Acid (PLA) by melt mixing to create a bio-degradable, mechanically reinforced nanocomposite filament. The filament was then processed by Fused Filament Fabrication (FFF) 3D-printer, and the morphology and characteristics before and after printing were compared. We found that addition of WS_2_-NT to PLA by extrusion mixing increases the elastic modulus, yield strength and strain-at-failure by 20%, 23% and 35%, respectively. Moreover, we found that the printing process itself improves the dispersion of WS_2_-NT within the PLA filament, and does not require changing of the printing parameters compared to pure PLA. The results demonstrate the advantage of WS_2_-NT as reinforcement specifically in 3D-printable polymers, over more traditional nano-reinforcements such as graphene and carbon nanotubes. WS_2_-NT based 3D-printable nanocomposites can be used for variety of applications from custom-made biodegradable scaffold of soft implants such as cartilage-based organs and biodegradable soft stents to the more general easy-to-apply nano-reinforced polymers.

## Introduction

Advanced manufacturing, and especially 3D printing, has attracted a lot of attention in the last decades, mostly due to its high versatility in materials and designs – including topographies that cannot be produced in traditional subtractive manufacturing, and the ease of custom-tailoring products. The latter has enormous advantage in the medical fields – especially orthopedic, dental and plastic medicine, as it allows precise anatomical design of the printed device – be it an implant, a surgical tool or a support model – to the specific patient^1,2^.

The most widely used printing technique is the Fused Filament Fabrication (FFF) used to fabricate thermoplastics such as Polylactic Acid (PLA), Acrylonitrile Butadiene Styrene (ABS) and Polycarbonate (PC). In FFF, a thermoplastic filament is extruded through a heated nozzle onto a build platform as layer by layer are infused together to a final solid shape. The temperature of the extruding nozzle and build platform, as well as layer thickness, orientation, and printing speed can be tailored to control the properties of the final product. The use of thermoplastic polymers allows for fast, low-cost and relatively simple fabrication of light-weight structures, but the final product will be mechanically weak. Moreover, as the demand for multifunctional materials increases, the need for complex designs to optimize the use of such materials also rises^3–5^.

Polymer matrix composites, and especially nanocomposites, propose an elegant solution: the micro- and nano-additives will provide improved mechanical^6^, electrical^7^, and other desired properties, while the polymer matrix maintains ease of fabrication and light-weight structure. The choice of nanofillers and thus of properties is vast^4,8^, but the transition from a pure polymer to polymer nanocomposite is far from trivial. Issues such as adhesion, orientation and dispersion of the nanofillers within the final product are still a challange^3^, as they affect not only the properties of the final product, but the printing parameters or even the very ability to print as well^7^. Moreover, while post-printing specimens have been examined^7,9^ and compared to their molded respectives^6^, no work has yet examined the effect of the printer itself, namely – compared what is going into the printer to what is coming out of it.

In this work, we present the preparation and printing of PLA/WS_2_- Nanotubes (WS_2_-NT). WS_2_-NT are metal dichalcogenides nanotubes of the shape MX_2_ (M = transition metal, Mo, W, etc.; X = S, Se, Te). In its tube form, WS_2_ is a semiconductor, nontoxic, and dispersible in both organic solvents and polymers. With a Young’s modulus of 160 GPa, bending modulus of 217 GPa, tensile strength between 16–20 GPa and strain at failure larger than 10%^10,11^, WS_2_-NT are a non-toxic alternative to Carbon Nanotubes (CNT)^12,13^.

**Figure 1:**
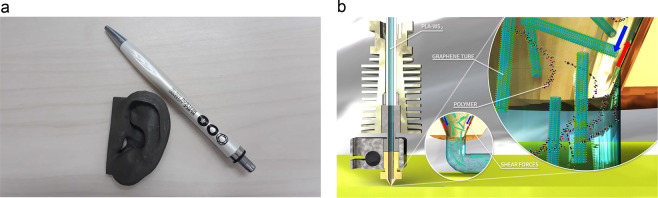
FFF-printing of PLA/ WS_2_-NT composite. (**a**) a model of a human ear (1:1), printed of PLA/ WS_2_-NT composite (with a pen to scale). Un-dyed, the model color is determined by the color of the WS_2_-NT. (**b**) illustration of the printing process.

Due to the biodegradability of PLA, and the biocompatibility of WS_2_-NT, PLA/WS_2_-NT is a biodegradable nanocomposite. Reinforced PLA is used in biodegradable scaffolds for tissue engineering^3,9,14^, and previous work on PLA/WS_2_-NT^15^ have demonstrated improved mechanical and rheological properties compared to that of pure PLA, with little change in the polymer viscosity. The preservation of viscosity together with the clear advantages of custom-tailored scaffolds and prostheses inspired a high motivation in adjusting PLA/WS_2_-NT composite for FFF. An example for possible application can be seen in Fig. 1a, as a printed model of a cartilage-based organ (in that case – an ear) is presented. To examine the effect of the FFF printing process itself (illustrated in Fig. 1b), the morphology and mechanical properties of the feeding filament were compared to those of the printed specimens.

## Results and Discussion

**Figure 2:**
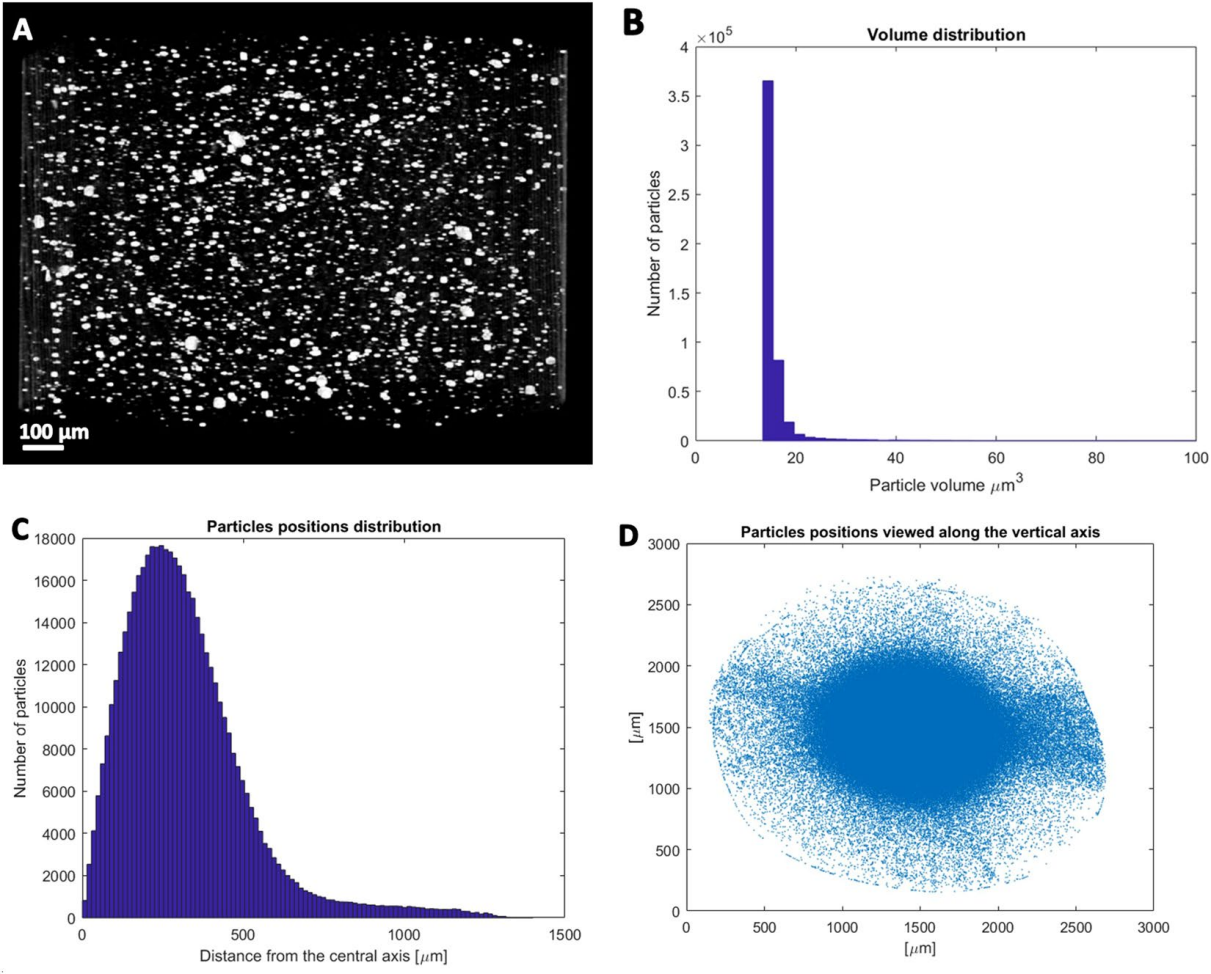
µ-XCT of pre-printed filament. (**A**) 3D rendering image of PLA filament with 0.5 wt % INT–WS_2_, (**B**) volume distribution of the nanotubes and aggregates in the PLA matrix, (**C**) particles positions distribution, (**D**) particles positions distribution along the vertical axis. Note the large concentration of the particles at the filament center. Images drawn in Avizo Software version 9.7. Link to the software can be found in Supplementary Information.

**Figure 3:**
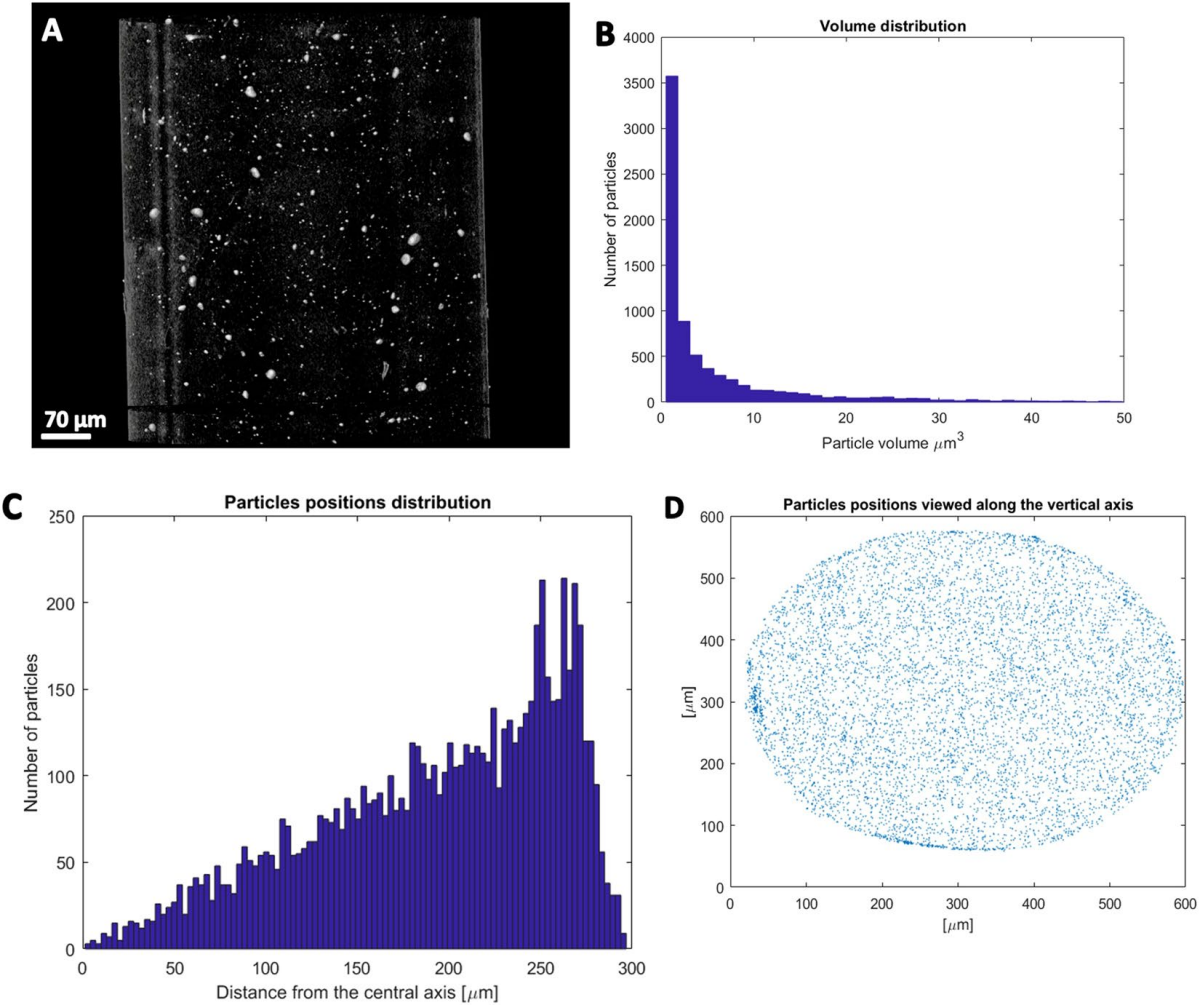
µ-XCT of post-printed filament. (**A**) 3D rendering image of PLA filament with 0.5 wt % INT–WS_2_, (**B**) volume distribution of the nanotubes in the PLA matrix, (**C**) particles positions distribution, (**D**) particles positions distribution along the vertical axis. Note that post-print particles are much more evenly distributed. Images drawn in Avizo Software version 9.7. Link to the software can be found in Supplementary Information.

First of all, the morphology of PLA/WS_2_-NT composite before and after printing was compared using micro-X-ray computerized tomography (µ-XCT). The contrast between the heavy atoms of the WS_2_-NT and the light carbon atoms in the polymer backbone allows for good separation between the materials even at the relatively low resolution (0.5 µm) of the instrument, in which individual nanotubes cannot be determined. Pre-printed filament (Fig. 2), with an average diameter of 2.35 mm, exhibits very few large agglomerates overall, but most of the nanotubes are concentrated at the filament center – showing uneven dispersion of the nanotubes throughout the filament diameter. In contrast, the 0.5 mm diameter post-printed filament (Fig. 3) exhibits even smaller WS_2_-NT clusters that are now evenly dispersed along the filament diameter, apart from what appears to be dust artifact condensed around the hot, semi-liquid filament surface during printing. Comparing the PLA/WS_2_-NT pre- and post-printing suggest that one of the main advantages in using FFF for nanocomposites processing is the improved dispersion of nanoparticles without the need for solvent-supported dispersion. For example, the biggest agglomerate decrease from 92.2 × 10^3^ µm^3^ (pre –printing filament) to 157.4 µm^3^ (post-printing filament). In addition, the post-printing filament presents smaller average particle volume compared to the pre-printing filament. The volume fraction of the nanotubes in the pre-printed filament as obtained by the µ-XCT analysis is 0.33 vol%, while in the post-printed filament it is 0.80 vol%. It should be noted that both volume fractions are higher than the expected value of about 0.2 vol% (0.5 wt%). This discrepancy can be also attributed to the limited resolution of the µ-XCT and the beam hardening during the data collection stage, which assigns a larger than expected volume for anisotropic objects, like nanotubes. Therefore, to further validate the preservation of WS_2_-INT in the filament, thermogravimetric analysis (TGA) was used to determine the weight mass of the nanotubes in the polymer matrix (see SI-1). The weight mass of the nanotubes was reduced from 1.27 wt% to 0.42 wt% for pre- and post-printed filaments, respectively. However, the weighing accuracy of TGA measurement is ±0.1%, which led to a percentage weight loss accuracy of ±0.5 wt%. Unfortunately, the percent weight loss accuracy of the TGA includes the nanotubes’ concentration in the PLA matrix. In addition, the difference in nanotubes’ concentration pre- and post-printed filaments is also within the percent weight loss accuracy. Therefore, the nanotubes’ concentration in the PLA matrix pre- and post-printed falls within instrument error. As the results of the TGA and µ-XCT are both within the margin of error and in opposite directions, we assumed it is the same.

**Figure 4:**
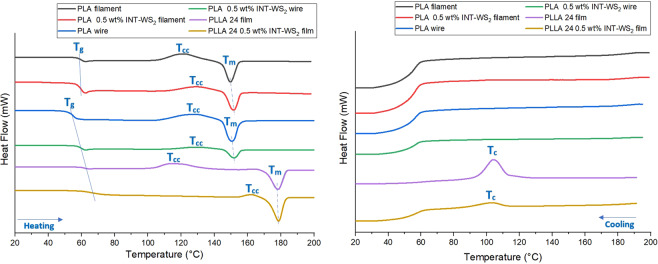
DSC Thermograms of (**A**) heating and (**B**) cooling PLA and PLA/ WS_2_-NT pre- and post-printed filament, compared to solvent-casted thermograms^15^.

Second, the thermal behavior of PLA with and without WS_2_-NT, pre- and post-printing, as measured by Dynamic Scanning Calorimetry (DSC) (Fig. 4) was used to calculate the glass transition, crystallization temperature and enthalpy, melting temperature and crystallinity percentage (Table 1). The results show negligible effect of the printing process on the composite crystallinity. It should be noted, that no changes were required in the printing parameters. Transition between the pure matrix and the nanocomposite in printing are thus simple and straight-forward.

**Table 1:** DSC parameters with standard deviation of PLA and PLA/ WS_2_-NT pre- and post-printed filament, compared to solvent-casted parameters^15^.

Method of Preparation	Sample	Tg(°C)	Tcc(°C)	ΔHcc(J/g)	Tm(°C)	ΔHm(J/g)	Tc(°C)	ΔHc(J/g)	%Cry(%)	1−λ(%)
3D printing	PLA filament	59.9 ± 0.5	121.4 ± 0.5	19.3 ± 2.5	149.5 ± 0.2	20.9 ± 1.5	91.1 ± 9.4	0.5 ± 0.1	1.7 ± 1.1	0.6 ± 0.1
	PLA with 0.5 wt% INT-WS_2_ filament	60.1 ± 0.1	128.8 ± 0.3	5.8 ± 0.3	151.5 ± 0.2	8.0 ± 0.6	99.3 ± 0.8	0.6 ± 0.1	2.3 ± 0.9	0.6 ± 0.1
	PLA wire	59.9 ± 0.1	128.8 ± 0.1	25.5 ± 23.4	150.9 ± 0.6	26.1 ± 23.9	96.2 ± 2.0	0.7 ± 0.5	0.6 ± 0.5	0.8 ± 0.6
	PLA with 0.5 wt% INT-WS_2_ wire	60.1 ± 0.4	129.4 ± 0.3	5.8 ± 0.4	151.7 ± 0.5	7.5 ± 0.6	104.1 ± 1.3	0.4 ± 0.0	1.8 ± 0.5	0.5 ± 0.0
Solvent casting^15^	PLLA 24	61.7 ± 0.4	114.8 ± 0.3	32.4 ± 12.0	178.6 ± 0.4	33.9 ± 12.2	101.6 ± 0.4	2.0 ± 0.1	1.6 ± 0.2	2.1 ± 0.4
	PLLA 24 with 0.5 wt% INT-WS_2_	65.5 ± 1.7	107.9 ± 7.9	3.1 ± 0.6	178.9 ± 0.3	37.9 ± 1.5	103.0 ± 0.9	30.6 ± 6.1	37.4 ± 2.1	32.9 ± 6.6

Comparing the DSC results to those of solvent-cast PLA and PLA/ WS_2_-NT^15^ shows that solvent-casting induces the highest degree of crystallinity, most likely due to the much slower process of solvent evaporation that allows for larger crystals to grow. It is worth noting that dedicated-for-printing off-the-shelf PLA can also reach over 30% crystallinity post-printing, so optimization of formulation might be helpful in achieving higher crystallinity for improved mechanical properties, or lower for faster degradation.

In FFF 3D printing, numerous parameters influence the mechanical properties of the printed object. The number of studies investigating the printing parameters is increasing day by day. The correlation of different patterns, layer height and layer orientation were tested. The one-way analysis of variance (ANOVA) of the storage modulus at 37 °C and 50 °C from dynamic mechanical analysis (DMA) measurement (see SI-2) confirms that each 3D parameter (layer height, pattern and orientation) is an individual factor. From the analysis of the tensile test results, the best appropriate parameters to printed PLA is with layer height of 150 µm, Zig-Zag pattern and 0° orientation.

**Figure 5:**
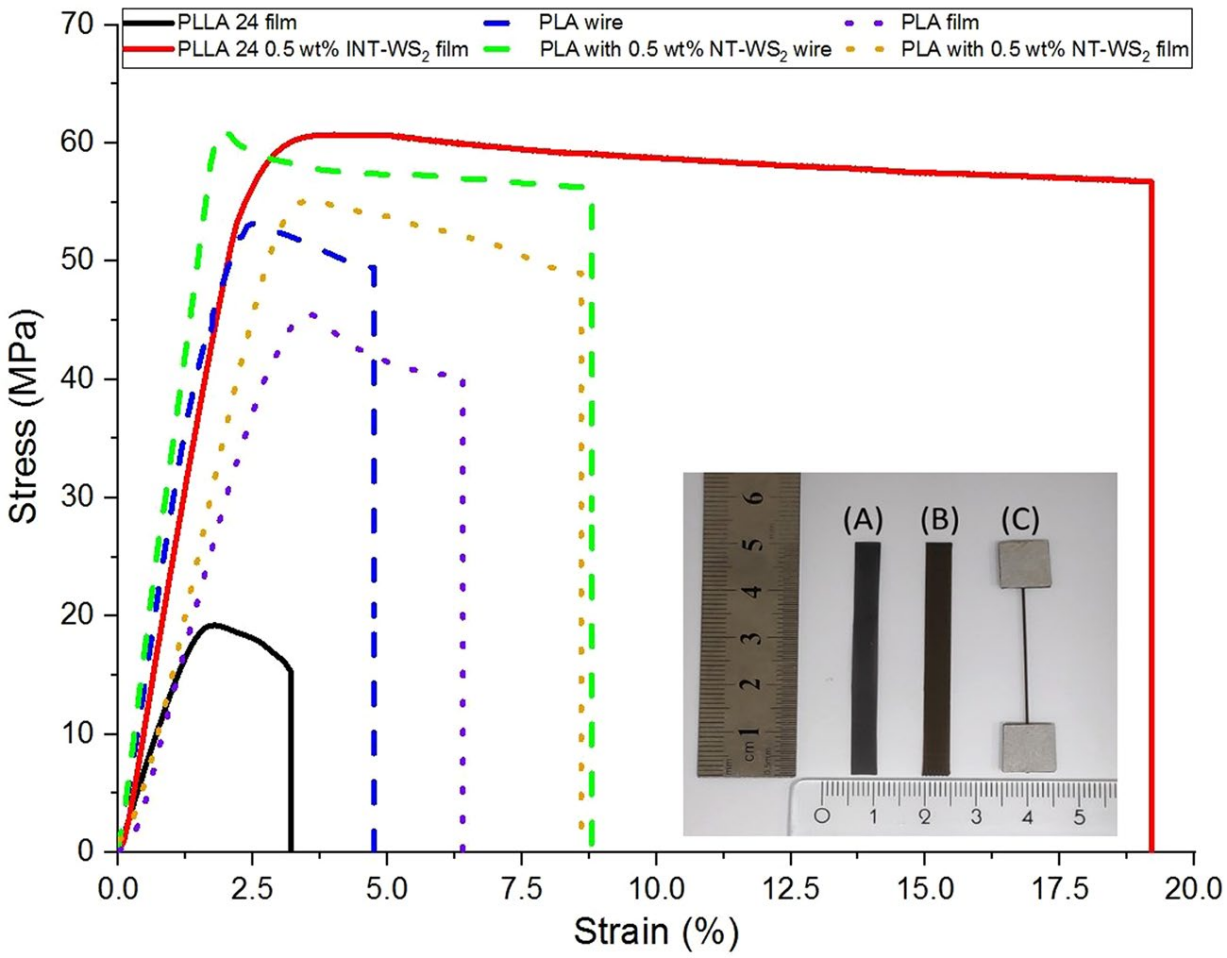
Stress-strain representing curves of PLA and PLA/ WS_2_-NT pre- and post-printed filament, compared to solvent-casted.^15^. Inset: (A) – cast film, (B)- printed film, (C) – printed wire. Rule-bar to scale.

The mechanical properties of printed PLA and PLA-WS_2_ – NT composite were also compared to those of solvent-cast films previously published^15^ (Fig. 5 and Table 2). As expected, the modulus and strength of PLA-WS_2_ are significantly larger than those of pure PLA. Interestingly, printed films exhibit lower values than printer-extracted wires, even though the printed films have the same orientation as the wires - parallel to the tensile axis. A fact that suggests adhesion problems between the printed layers. Such problems could perhaps be solved by post-processing treatment.

**Table 2:** Mechanical properties with standard deviation of PLA and PLA/ WS_2_-NT pre- and post-printed filament, compared to solvent-casted properties^15^.

Method of Preparation	Sample	Modulus (GPa)	Yield Strength (MPa)	Strain at failure (%)	Toughness (MPa*%)	Length (mm)	Thickness (mm)	Diameter (mm)
Solvent casting^15^	PLLA 24 film	1.4 ± 0.05	22.0 ± 4.1	3.5 ± 0.6	1.2 ± 0.3	30.0 ± 0.1	0.14 ± 0.1	—
	PLLA 24 film with 0.5 wt% INT-WS_2_	2.8 ± 0.2	60.9 ± 2.8	18.4 ± 5.2	5.7 ± 0.7	30.0 ± 0.1	0.10 ± 0.1	—
3D printing	PLA wire	3.6 ± 0.4	52.8 ± 10.5	4.8 ± 2.8	1.6 ± 1.1	34.1 ± 0.3	—	0.5 ± 0.0
	PLA with 0.5 wt% INT-WS_2_ wire	4.2 ± 0.5	63.2 ± 10.1	8.8 ± 7.5	4.7 ± 4.1	33.9 ± 1.1	—	0.5 ± 0.0
	PLA film	1.82 ± 0.17	46.76 ± 3.82	6.37 ± 1.95	2.11 ± 0.91	30.0 ± 0.1	0.22 ± 0.01	—
	PLA with 0.5 wt% INT-WS_2_ film	2.19 ± 0.15	57.56 ± 4.83	8.63 ± 3.21	3.84 ± 1.78	30.0 ± 0.1	0.15 ± 0.01	—

Printed films of pure PLA outperform their solvent-cast respective in all parameters – modulus, strength, strain-at-failure and toughness. Thus, the results confirm the strong disadvantage of solvent-casting, namely – residues of solvent acting as plasticizers in the polymer. As extrusion and FFF do not require solvent – the mechanical properties improve. However, the gap is inverted, with higher modulus and strain-at-failure for the solvent-cast films (strength and toughness are similar) when WS_2_ nanotubes are introduced to the composite. The most reasonable explanation to the favorable properties of the PLA/ WS_2_-NT solvent-cast film is its increased crystallinity, as confirmed by DSC. Such increased crystallinity due to WS_2_-NT addition was not observed in the fast-solidifying printed PLA/ WS_2_-NT, and so lower crystallinity also results in lower modulus, strength and toughness (see inset in Fig. 5 - specimens picture). The improved mechanical properties might also suggest better dispersion of the WS_2_ INT by solvent-casting, countering the solvents-induced deterioration of the polymer matrix itself. As showed in previous work^15^, differences in dispersion can account to up to 50% difference in strength, 20% in modulus and 80% in toughness, as befitting the difference between printed and cast PLA/ WS_2_-NT. The difference in dispersion can also explain the large error range of the printed PLA/ WS_2_-NT wires. In addition, in both techniques, the WS_2_-NT with 0.5 wt% was able to improve all the mechanical properties of the neat PLA, while other studies have failed to resolve all the mechanical properties of the PLA reinforced matrix^16–18^.

Unlike solvent casting, 3D printing allows the fabrication of many forms, including complex shapes and standard geometries such as dog-bones specimens. The printed pure PLA exhibits slightly higher modulus values but twice lower strength and strain at failures values compared to off the shelf printed PLA with same printing orientation - parallel to the tensile axis.^19,20^ The addition of WS_2_-NT further increases all of the mechanical properties of the composite – more so than in printed films (see SI-3). This enhanced reinforcement of thicker specimens is encouraging, as it supports the possibility of using printed PLA/ WS_2_-NT for life-size scaffolds, such as demonstrated above.

## Conclusions

We have demonstrated, for the first time, the dispersion of WS_2_-NT in PLA thermoplastic via melt extrusion, followed by FFF printing. The results show no changes in the thermal properties of the melted composite compared to the neat polymer, and therefore no changes required in the printing parameters – indicating ease of WS_2_-NT implementation in materials for FFF. Such implementation is to be desired, as WS_2_-NT presence enhances the mechanical properties of printed PLA such as modulus, yield strength, elongation at failure and toughness.

Moreover, after FFF processing, the dispersion of the WS_2_-NT in the matrix has improved compared to pre-printed filament. When compared to their solvent-cast equivalent, the printed polymer preserves its mechanical integrity better, but the combination of the slow solidification and WS_2_-NT as crystal-nucleation sites increase crystallinity in the solvent-casted composite and thus its modulus.

These results show both the possibility and the advantage of FFF printed PLA/ WS_2_-NT composite. The processing flexibility allows for various shapes, including medical uses such as cartilage scaffolds and joints.

## Experimental

WS_2_ nanotubes (INT-WS_2_) 1–20 micron long with a diameter of 30–150 nm were purchased from Prof. A. Zak, Holon Institute of Technology, Israel. The synthesis of the INT-WS_2_ was according to the protocol described in the literature review. PLA pellets (4032D grade, NatureWorks) with a density of 1.24 g/cm3, were purchased from ReprapWorld, Netherlands.

To prepare the nanocomposite filament, PLA pellets (5 kg) and WS_2_-INT (12.5 g) were first dried in a vacuum oven at 50 °C overnight before processing. Then, PLA pellets were mixed with 0.5 wt% of WS_2_-INT in the melt state using a lab-scale co‐rotating twin screw extruder (EUROLAB Digital 16 ‘Prism’, D = 16 mm, L/D = 24) operated at 160 °C and 60 rpm. The melted compound was continuously cooled down to solidify under cold water at 10 °C, then pelletized – creating PLA/WS_2_-INT composite pellets. Pellets of either neat PLA or PLA/WS_2_-INT composite were processed into filaments of the desired diameter (2.85 mm) by extrusion at 160 °C and 60 RPM followed by water cooling at 10 °C.

PLA and PLA/WS_2_-INT filaments (diameter of 2.85 mm and 2.35 mm, respectively) were printed using a Sigma R19 FFF printer (BCN3D Technology, Barcelona, Spain). Brass nozzles with 0.4 mm diameter were used with the printed temperature at 205 °C and platform temperature at 55 °C. The printing head speed was set to 60 mm/s with layer height of 0.15 mm and Zig-Zag pattern. Specimens were printed with no shell layer and the infill was set to 100%. Every printed specimen was sliced to G-code using BCN3D Cura 1.1.0 software. The samples were printed to films with 0° orientation (parallel to the test axis). The dimensions of the printed films: 5 mm wide and 50 mm long, to allow gauge length test at 30 mm. Also, wires with an average diameter of 0.5 mm were tested with a gauge length of 30 mm.

The dispersion of the nanotubes in the PLA matrix was imaged with a Zeiss Xradia 520 Versa (Zeiss X-ray Microscopy, Pleasanton, CA, USA), under working conditions of X-ray source voltage (100 kV) and current (90 µA). Image processing and analysis were done with the Avizo software. The 2.35 mm diameter filament was imaged at 0.36 µm voxel size, while the 0.5 mm filament was imaged at 0.81 µm voxel size. The images were corrected for beam hardening.

Thermogravimetric analysis (TGA) measurements were carried out using a TGA-IR Q5000 IR system. Pre and post PLA and PLA/WS_2_-INT filaments samples were analyzed in nitrogen atmosphere, from 40 °C to 500 °C, with heating rate of 10 °C/min.

Thermal characterization was performed with DSC 250 (TA Instruments, New Castle, DE, USA). Both PLA and PLA with 0.5 wt% INT-WS_2_ were measured before and after printing, and compared to their respective solvent-cast, as previously published^15^. Temperature and enthalpy calibrations were performed using indium. Samples 5 mg in weight were placed in an aluminum pan and measured against an empty pan as a reference. Measurements were carried out under 50 mL/min nitrogen flow rate according to the following protocol: First, heating from 30 to 200 °C and 3 min at 200 °C (in order to erase the thermal history). Then, cooling down to 30 °C, and, finally, a 2nd heating until 200 °C. All scans were performed at 10 C/min under 50 mL/min nitrogen flow rate. From the mid-point of the (heating scan) thermograms, the glass transition (Tg), cold crystallization (Tcc), and melting (Tm) temperatures were determined. The crystallization temperature (Tc) was determined from the cooling scan. The degree of crystallinity was calculated from the DSC curves in two ways:

(1) *Xc =* (Δ*H*_*m*_ − Δ*H*_*cc*_)/Δ*H°*_m_ ×100%

for heating^21^, and

(2) (1 −*λ*) = Δ*H*_*c*_/Δ*H°*_m_

for cooling^22^.

ΔH_m_, ΔH_cc_ (heating), and ΔH_c_ (cooling) are the melting enthalpy, cold crystallization enthalpy, and crystallization enthalpy (J/g), respectively; ΔH_m_° is the heat of fusion for completely crystallized PLA (93 J/g)^23^.

Mechanical tests on both extracted wires and printed films were performed using Instron-5944 (Instron, Norwood, MA, USA) equipped with a 10 N load-cell at room temperature and a stretching speed of 1 mm/min. The load and displacement were recorded by dedicated software provided by the manufacturer (Bluehill3, Norwood, MA, USA). Dog-bone samples (ASTM D638 - Type IV) were 3D printed and tested using MTS- 20/M tensile testing machine equipped with a 100 kN load-cell at room temperature and a stretching speed of 5 mm/min. Fifteen specimens of each type were tested, and the results are given as average values. The load and displacement were recorded by dedicated software provided by the manufacturer (TestWorks, Eden Prairie, Minnesota, USA).

## Supplementary information

Supplementary information.

## References

[1] Visser, J., Melchels, F. P. W., Weinans, H., Kruyt, M. C. & Malda, J. [Applications of 3D printing in medicine; 5 years later]. *Ned. Tijdschr. Geneeskd*. **163** (2019). 31166096

[2] Mathew Essyrose, Domínguez-Robles Juan, Stewart Sarah A., Mancuso Elena, O’Donnell Kieran, Larrañeta Eneko, Lamprou Dimitrios A. (2019). Fused Deposition Modeling as an Effective Tool for Anti-Infective Dialysis Catheter Fabrication. ACS Biomaterials Science & Engineering.

[3] Ngo TD, Kashani A, Imbalzano G, Nguyen KTQ, Hui D (2018). Additive manufacturing (3D printing): A review of materials, methods, applications and challenges. Compos. Part B Eng..

[4] Wang X, Jiang M, Zhou Z, Gou J, Hui D (2017). 3D printing of polymer matrix composites: A review and prospective. Compos. Part B Eng.

[5] Lee J-Y, An J, Chua CK (2017). Fundamentals and applications of 3D printing for novel materials. Appl. Mater. Today.

[6] Dul S, Fambri L, Pegoretti A (2016). Fused deposition modelling with ABS-graphene nanocomposites. Compos. Part A Appl. Sci. Manuf.

[7] Gnanasekaran K (2017). 3D printing of CNT- and graphene-based conductive polymer nanocomposites by fused deposition modeling. Appl. Mater. Today.

[8] Farahani Rouhollah D., Dubé Martine, Therriault Daniel (2016). Three-Dimensional Printing of Multifunctional Nanocomposites: Manufacturing Techniques and Applications. Advanced Materials.

[9] Angjellari M (2017). Beyond the concepts of nanocomposite and 3D printing: PVA and nanodiamonds for layer-by-layer additive manufacturing. Mater. Des.

[10] Kaplan-Ashiri I (2006). On the mechanical behavior of WS_2_ nanotubes under axial tension and compression. PNAS.

[11] Rozenberg BA, Tenne R (2008). Polymer-assisted fabrication of nanoparticles and nanocomposites. Prog. Polym. Sci..

[12] Pardo M, Shuster-Meiseles T, Levin-Zaidman S, Rudich A, Rudich Y (2014). Low cytotoxicity of inorganic nanotubes and fullerene-like nanostructures in human bronchial epithelial cells: Relation to inflammatory gene induction and antioxidant response. Environ. Sci. Technol..

[13] Fojtů M, Teo WZ, Pumera M (2017). Environmental impact and potential health risks of 2D nanomaterials. Environ. Sci. Nano.

[14] Ligon SC (2017). Polymers for 3D Printing and Customized Additive Manufacturing. Chem. Rev..

[15] Shalom Hila, Sui XiaoMeng, Elianov Olga, Brumfeld Vlad, Rosentsveig Rita, Pinkas Iddo, Feldman Yishay, Kampf Nir, Wagner H.D., Lachman Noa, Tenne Reshef (2019). Nanocomposite of Poly(l-Lactic Acid) with Inorganic Nanotubes of WS2. Lubricants.

[16] Wu JH, Yen MS, Kuo MC, Chen BH (2013). Physical properties and crystallization behavior of silica particulates reinforced poly(lactic acid) composites. Mater. Chem. Phys..

[17] Robles E, Urruzola I, Labidi J, Serrano L (2015). Surface-modified nano-cellulose as reinforcement in poly(lactic acid) to conform new composites. Ind. Crops Prod.

[18] Chieng BW, Ibrahim NA, Wan Yunus WMZ, Hussein MZ, Silverajah VSG (2012). Graphene Nanoplatelets as Novel Reinforcement Filler in Poly(lactic acid)/Epoxidized Palm Oil Green Nanocomposites: Mechanical Properties. Int. J. Mol. Sci..

[19] Lanzotti A, Grasso M, Staiano G, Martorelli M (2015). The impact of process parameters on mechanical properties of parts fabricated in PLA with an open-source 3-D printer. Rapid Prototyp. J.

[20] Chacón JM, Caminero MA, García-Plaza E, Núñez PJ (2017). Additive manufacturing of PLA structures using fused deposition modelling: Effect of process parameters on mechanical properties and their optimal selection. Mater. Des.

[21] Ero-Phillips, O., Jenkins, M. & Stamboulis, A. Tailoring Crystallinity of Electrospun Plla Fibres by Control of Electrospinning Parameters. *Polymers (Basel)*. **4**, 1331–1348 (2012). Eq. #2

[22] Naffakh, M., Marco, C. & Ellis, G. Development of novel melt-processable biopolymer nanocomposites based on poly(L-lactic acid) and WS2 inorganic nanotubes. *CrystEngComm* **16**, 5062–5072 (2014). Eq.#1

[23] Södergård A, Stolt M (2002). Properties of lactic acid based polymers and their correlation with composition. Progress in Polymer Science (Oxford).

