# Supplementary material for: Strong, tough and bio-degradable polymer-based 3D-ink for fused filament fabrication (FFF) using WS2 nanotubes
Source: Sci Rep. 2020 Jun 1;10:8892. doi: 10.1038/s41598-020-65861-w (PMC7264276; doi:10.1038/s41598-020-65861-w)
Supplement: Supplementary file 1 — Supplementary information. [file 41598_2020_65861_MOESM1_ESM.docx]

**Strong, tough and bio-degradable polymer-based 3D-ink for fused filament fabrication (FFF) using inorganic WS_2_ nanotubes.**

Hila Shalom^1,2^, Sergey Kapishnikov^3^, Vlad Brumfeld^3^, Naum Naveh^4^, Reshef Tenne^2^, Noa Lachman^1*^

1. Department of Materials Science and Engineering, Faculty of Engineering, Tel-Aviv University, Ramat Aviv, Tel Aviv 6997801, Israel; [hilashalom@mail.tau.ac.il](mailto:hilashalom@mail.tau.ac.il)
2. Department of Materials and Interfaces, Weizmann Institute, Rehovot 76100, Israel; [reshef.tenne@weizmann.ac.il](mailto:reshef.tenne@weizmann.ac.il)
3. Department of Chemical Research Support,Weizmann Institute, Rehovot 76100, Israel; sergey.kapishnikov@weizmann.ac.il, brumfeldvlad@gmail.com
4. Polymers and Plastics Engineering Department, Shenkar College of Engineering, Design and Art, Ramat-Gan, Israel; naumn@shenkar.ac.il

* Correspondence: noala@tauex.tau.ac.il; Tel.: +972-3-640-6993

SI 1: TGA curves for PLA/WS_2_-NT pre- and post-printed filaments.


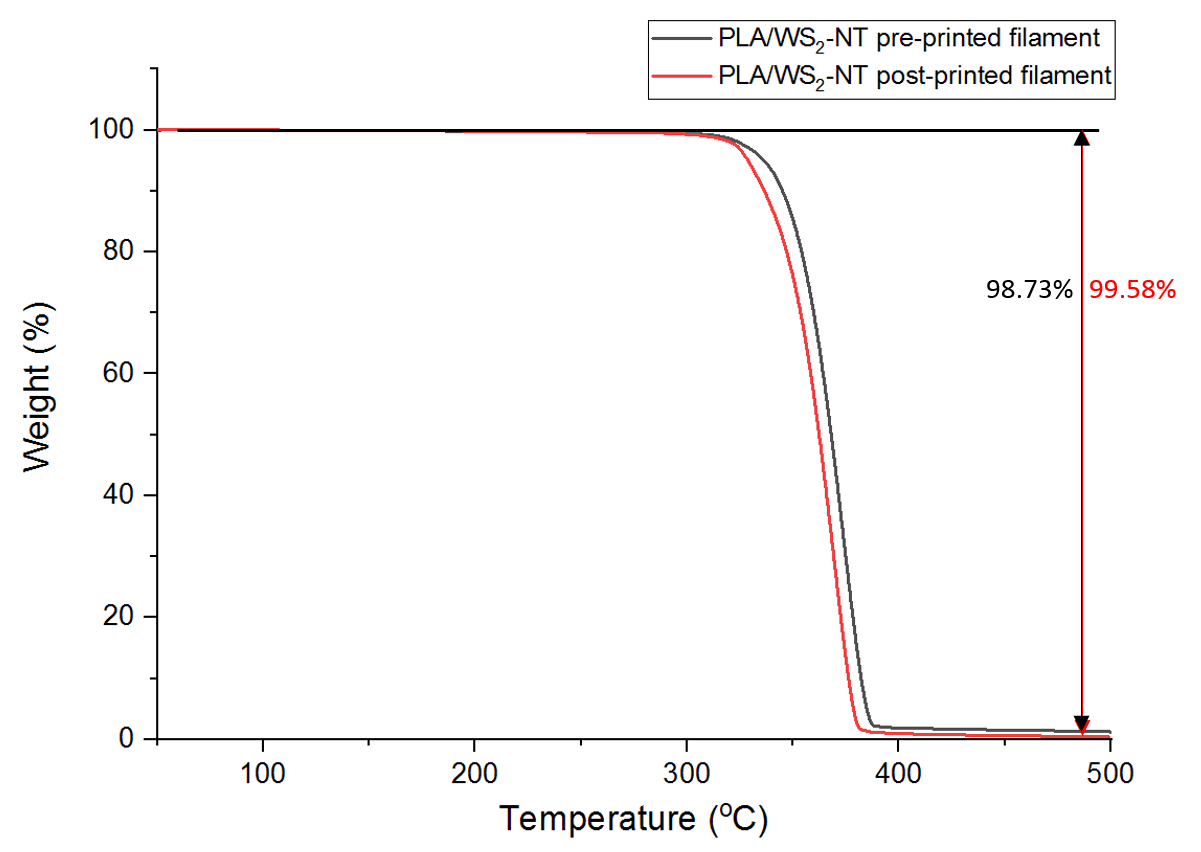


Figure SI1: TGA curves for PLA/WS_2_-NT pre- and post-printed filaments.

SI 2: printed PLA with different parameters tests, discussion and results:

Dynamic mechanical analysis (DMA) of rectangular samples (were 55 mm x 12.8 mm x 3.2 mm) was measured using DMA Q800 (TA Instruments). The five different specimens were tested in a dual cantilever mode by heating the samples at a rate of 1◦C/min with an oscillating amplitude of 15 µm and a frequency of 1, 10 and 100 Hz.

Commercial PLA (yellow PLA from Filaform, Australia) was printed to dog-bone samples (ASTM D638 - Type IV) using a Sigma R19 FFF printer (BCN3D Technology, Barcelona, Spain). Specimens were printed with different layer height (0.08, 0.1 0.15 and 0.3 mm), pattern (Zig-Zag and lines) and orientation (0° and 90°). The mechanical properties were measured using ‎Instron-5582 equipped with a 100 N load-cell at room temperature and a stretching speed of ‎‎0.25 mm/min. The loading framework and the ‎displacement were controlled by Bluehill's operating scheme.

DMA measurement was performed on printed commercial PLA with Zig-Zag and Lines patterns in two orientations (0° and 90°) and different layer heights (80, 100, 150 and 300 µm). In order to know whether the various print parameters (pattern, orientation and layer height) are affected by each other or not, one-way analysis of variance (ANOVA) was performed on the storage modulus of the printerd specimens at 37 °C and 50 °C (Figure SI1 and Table SI1). In both temperatures (37 °C and 50 °C), the P-value of the F-test is less than 0.05 and the *F*-ratio is higher compared to the P-value. Hence, the ANOVA analysis approving that each 3D parameter (layer height, pattern and orientation) is an individual factor.


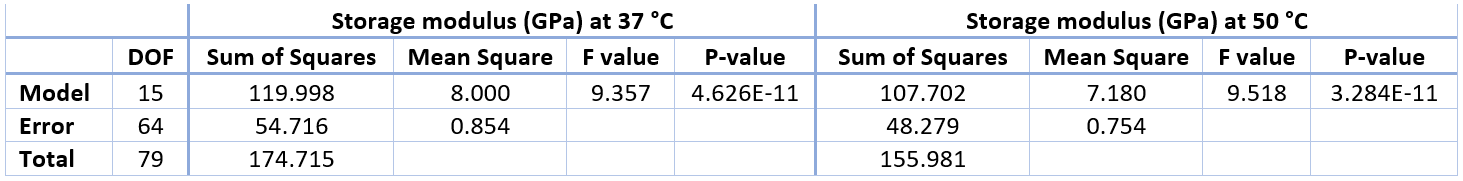
 Table SI2: Results of the ANOVA analyses for printed PLA specimens.

Figure SI2: ANOVA one-way results showing in box charts of storage modulus vs layer height, pattern and orientation at 37 °C (A) and 50 °C (B).


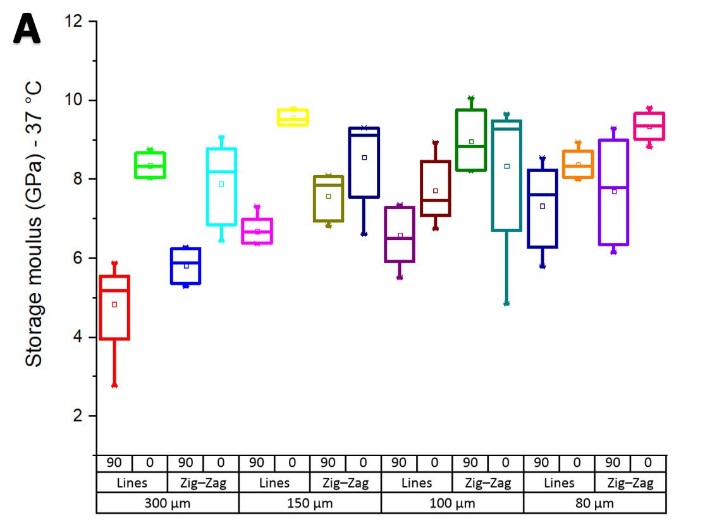

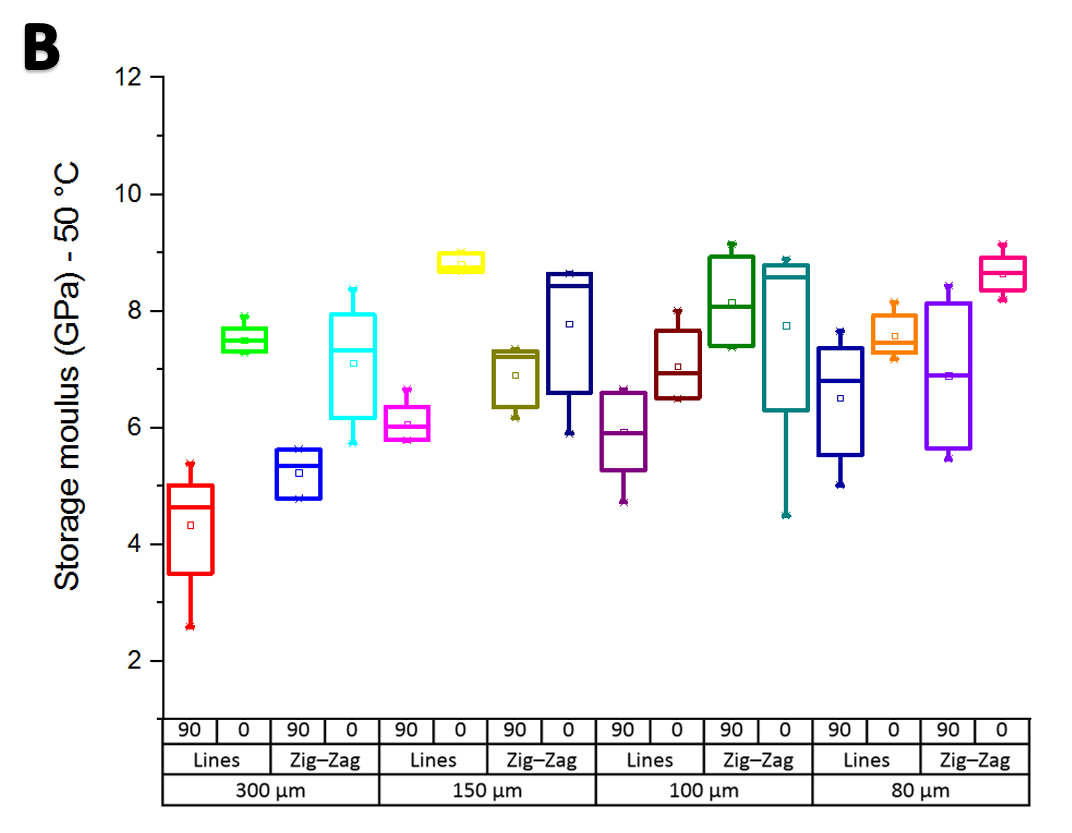


Printed layer height of 80 µm and 300 µm in both temperatures presents larger error ranges compared to the 100 µm and 150 µm layer heights. Printed layer height of 300 µm provides a very low printing resolution, which affects the lack of repeatability of the measured isotropic properties. In contrast, printing in low layer height (80 µm), provides high-resolution printing, but also increases anisotropy and produce storage modulus values with bigger error ranges compared to the rest of the results. Hence, these results also show the lack of repeatability of isotropic properties. Results of the printed layer height of 100 µm do not show repeatability between the Lines and Zig-Zag patterns with 0° and 90° orientations compared to the rest of the results. Therefore, another test performed on the printed object with a layer height of 150 µm and with different pattern and printed orientations, was the tensile test (Figure SI2 and Table SI2).

Zig-Zag pattern with 90° orientation presents the smallest elongation, this could be indicative of low adhesion between the stripes in the specimen. Also, Zig-Zag and Lines patterns with 0° orientation present the same strain at failure but different values of stress. However, the Zig-Zag pattern shows elastic material behavior, due to the presence of plastic deformation before breaking (Figure SI2), while the Lines pattern shows behavior of a brittle material, due to the absence of plastic deformation. The weaker specimen is Lines patterns with 90° orientation (maximum tensile stress of 4.68 MPa), also shows behavior of a brittle material.

| Sample | Young Modulus  (GPa) | Yield stress  (MPa) | Maximum tensile stress (MPa) | Strain at failure  (%) | Toughness (MPa)*(%) |
| --- | --- | --- | --- | --- | --- |
| 150 µm Zig-Zag 0° | 2.23 | 1.19 | 8.47 | 0.50 | 0.02 |
| 150 µm Zig-Zag 90° | 1.89 | 1.28 | 4.05 | 0.31 | 0.01 |
| 150 µm Lines 0° | 2.14 | 1.56 | 10.35 | 0.50 | 0.03 |
| 150 µm Lines 90° | 0.9 | 0.86 | 4.68 | 0.57 | 0.02 |

Table SI-3: Mechanical properties obtain from the Stress – Strain curves.

Figure SI3: Stress - strain curves of PLA with 150 µm layer height, different pattern (Lines and Zig-Zag) and orientation (0° and 90°).


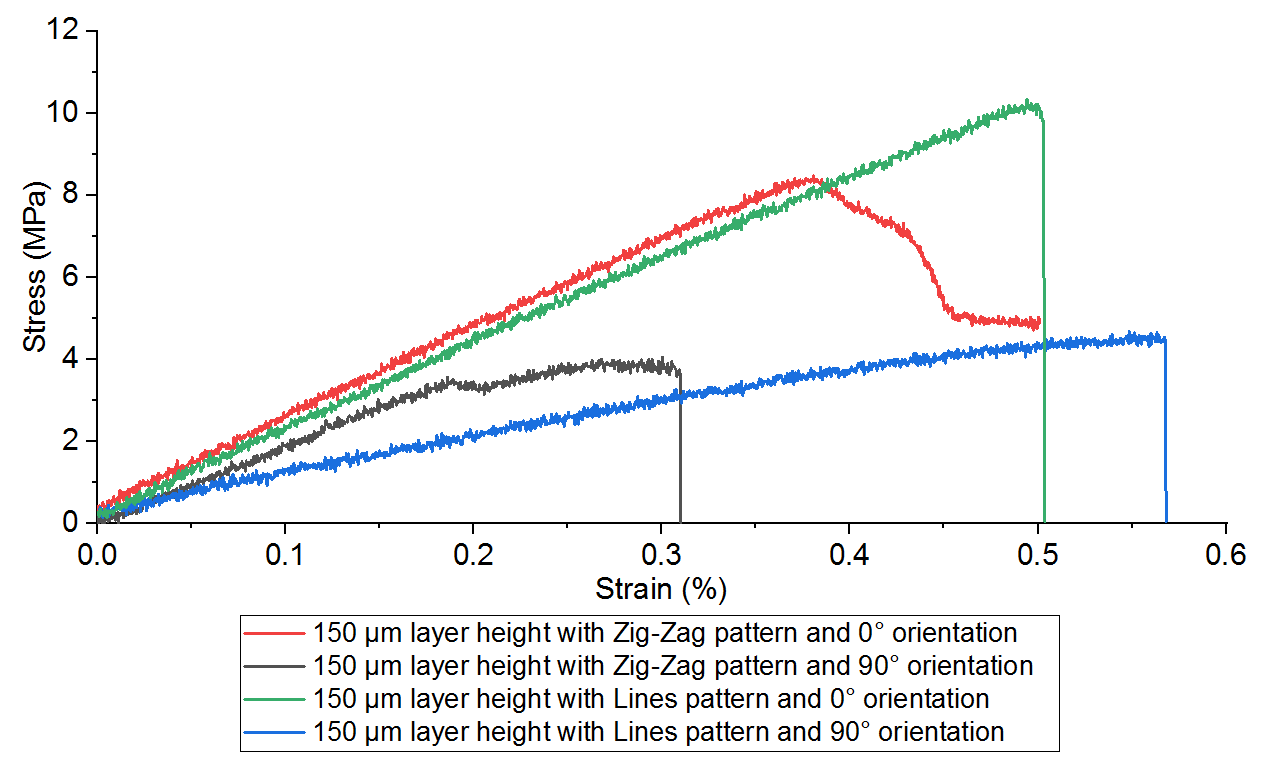


SI 3: Dog-bone mechanical tests and results:

Dog-bone samples (ASTM D638 - Type IV) of PLA and PLA/WS_2_-NT were 3D printed and tested using MTS- 20/M tensile testing machine equipped with a 100 kN load-cell at room temperature and a stretching speed of 5 mm/min. 15 specimens of each type were tested (one typical curve is shown in Figure SI1 for each composition), and the results were given as average values. The load and displacement were recorded by dedicated software provided by the manufacturer (TestWorks, Eden Prairie, Minnesota, USA).


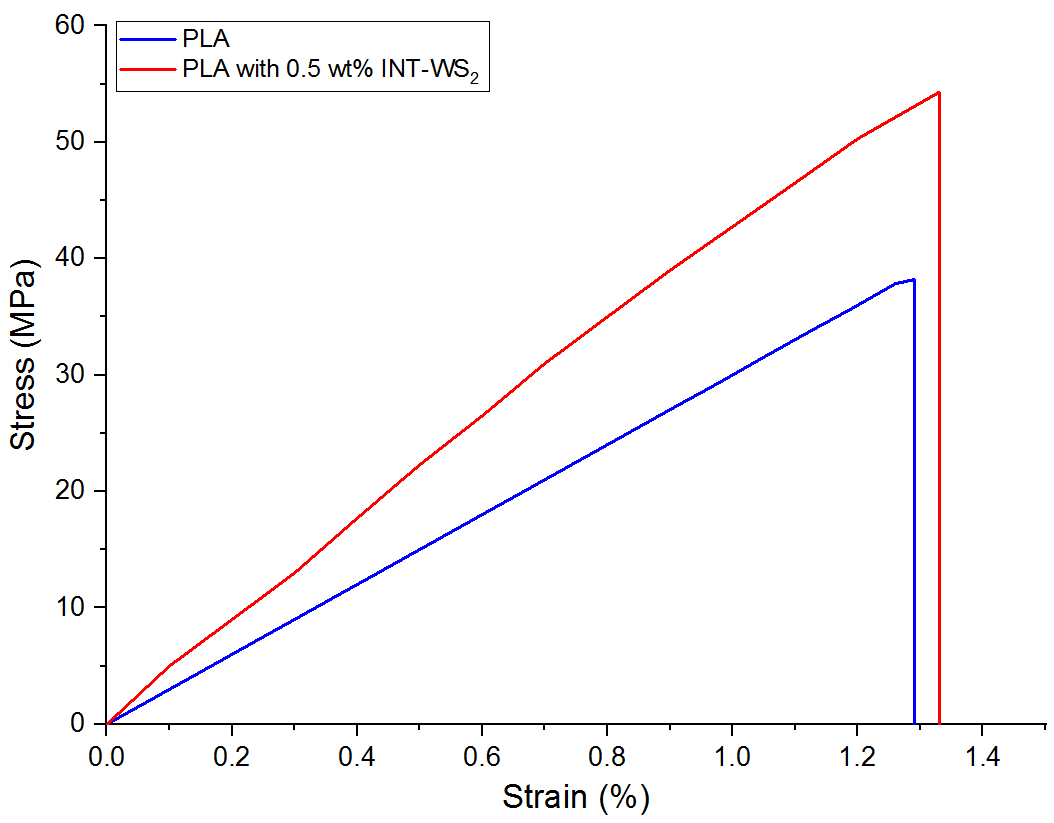


Figure SI4: Stress-strain representing curves of PLA and PLA/ WS2-NT printed dog-bones.

| Sample | **Modulus  (GPa)** | **Yield Strength (MPa)** | **Strain at failure**  **(%)** | **Toughness  (MPa*%)** | **Width**  **(mm)** | **Thickness (mm)** |
| --- | --- | --- | --- | --- | --- | --- |
| PLA | 3.37 ± 0.48 | 34.9 ± 7.08 | 1.29 ± 0.34 | 2.55 ± 1.11 | 3.46 ± 0.14 | 5.8 ± 0.38 |
| PLA 0.5 wt% INT-WS_2_ | 4.45 ± 0.31 | 49.9 ± 5.84 | 1.33 ± 0.21 | 3.63 ± 0.97 | 3.28 ± 0.05 | 5.9 ± 0.36 |

Table SI4: Mechanical properties of PLA and PLA/ WS2-NT printed dog-bones

SI 4: Link to Avizo Software version 9.7:

https://www.thermofisher.com/il/en/home/industrial/electron-microscopy/electron-microscopy-instruments-workflow-solutions/3d-visualization-analysis-software/avizo-materials-science.html
